# Protocol for fabricating electroless nickel immersion gold strain sensors on nitrile butadiene rubber gloves for wearable electronics

**DOI:** 10.1016/j.xpro.2021.100832

**Published:** 2021-09-15

**Authors:** Sara S. Mechael, Yunyun Wu, Yiting Chen, Tricia Breen Carmichael

**Affiliations:** 1 Department of Chemistry and Biochemistry, University of Windsor, Windsor, ON N9B3P4, Canada

**Keywords:** Physics, Chemistry, Material sciences

## Abstract

This protocol describes the fabrication of patterned conductive gold films on nitrile butadiene rubber (NBR) gloves for wearable strain sensors using electroless nickel immersion gold (ENIG) plating, a solution-based metallization technique. The resulting NBR/ENIG films are strain sensitive; resistance measurements of a patterned sensing array can be used to map human hand motions. This protocol also describes challenges related to the ENIG process and troubleshooting steps to achieve conformal gold films for strain sensing over a large working range.

For complete details on the use and execution of this protocol, please refer to Mechael et al. (2021).

## Highlights

• Detailed protocol to prepare strain sensors on nitrile butadiene rubber (NBR) gloves
• Procedure to activate NBR for electroless nickel immersion gold (ENIG) metallization
• Patterned ENIG deposition by airbrushing a commercially available latex emulsion
• Tensile strain increases the resistance of the ENIG film for wearable motion sensing

## Graphical abstract

**Figure:**
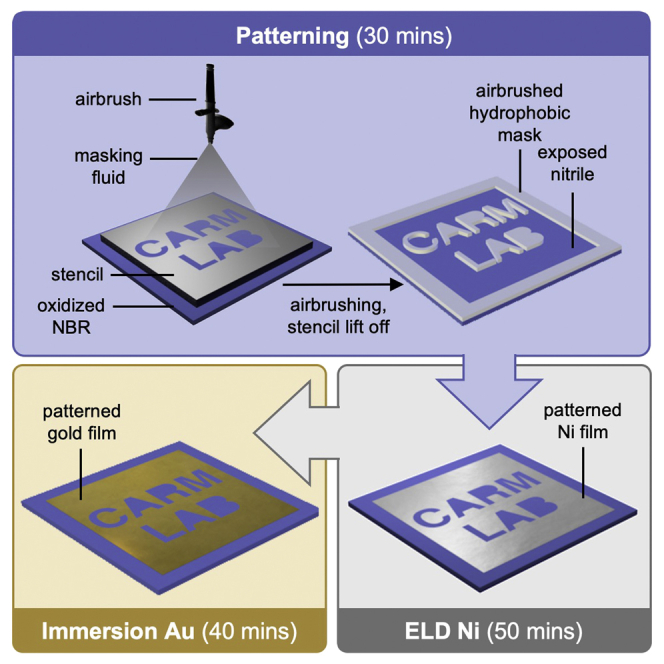

## Before you begin

The protocol below describes the specific steps for electroless nickel immersion gold (ENIG) plating on nitrile butadiene rubber (NBR) gloves to fabricate strain sensing arrays for human motion detection. ENIG is a scalable, low temperature, solution-based gold deposition technique that encompasses two solution-based plating steps: the electroless deposition (ELD) of a nickel film followed by an immersion gold process that galvanically displaces nickel with gold from a gold cyanide salt solution (Accogli et al., 2020). ENIG is a reliable additive manufacturing method used to deposit corrosion-resistant gold layers in printed circuit board (PCB) manufacturing (Accogli et al., 2020). We have previously applied this versatile process to prepare gold films on a variety of substrates such as NBR (Mechael et al., 2021), polydimethylsiloxane (Chen et al., 2019), and various textiles (Wu et al., 2018, 2020a, 2020b) for stretchable and wearable electronics. One advantage of this deposition method over physical vapor deposition (PVD) techniques is the ability to conformally deposit gold films on complex interior geometries because of the non-line-of-sight nature of solution plating. For example, our research group has applied ENIG deposition on polyester textiles to prepare conformal gold coatings that individually coat each fiber while preserving the void space between fiber bundles to maintain the wearability of the final e-textile (Wu et al., 2018). The benefit of conformal deposition also extends to the metallization of substrates with high surface roughness such as NBR gloves (Mechael et al., 2021). Here, we describe how to use ENIG to directly deposit sensing arrays on off-the-shelf NBR gloves by patterning the metal deposition on the joint locations of the hand (Mechael et al., 2021). NBR gloves are made of a copolymer of acrylonitrile and butadiene and have a high surface roughness. ENIG deposits uniform, conformal coatings on NBR gloves with a low sheet resistance of 3.1 ± 0.6 Ω/sq (Mechael et al., 2021).

Human hands are critical to the execution of a wide range of daily tasks involving fine and gross motor movements. Low-profile sensing arrays housed in a glove format can mine gesture data to guide therapeutic treatments, transcribe sign language or music, or interface with virtual reality (Chossat et al., 2015; Lee et al., 2015; O’Connor et al., 2017). Using off-the-shelf, disposable NBR laboratory gloves as a platform for resistive thin-film sensing arrays provides a ready-to-wear solution to monitor hand-specific gestures. The intrinsic roughness of the NBR glove facilitates a microcracking pattern in the ENIG film during deformation caused by bending a finger (Mechael et al., 2021). The formation of microcracks relieve the material of strain while simultaneously causing a measurable increase in the resistance of the film correlated to the magnitude of the strain. By depositing an array of sensors on the joint locations of the glove, each sensor contributes local information about the degree of bending at that joint. The amalgamation of information contributed by each sensor illustrates a full map of the position of the wearer’s hand that can be used in real-time applications such as gesture differentiation or robotic control (Mechael et al., 2021).

**Scheme 1:**
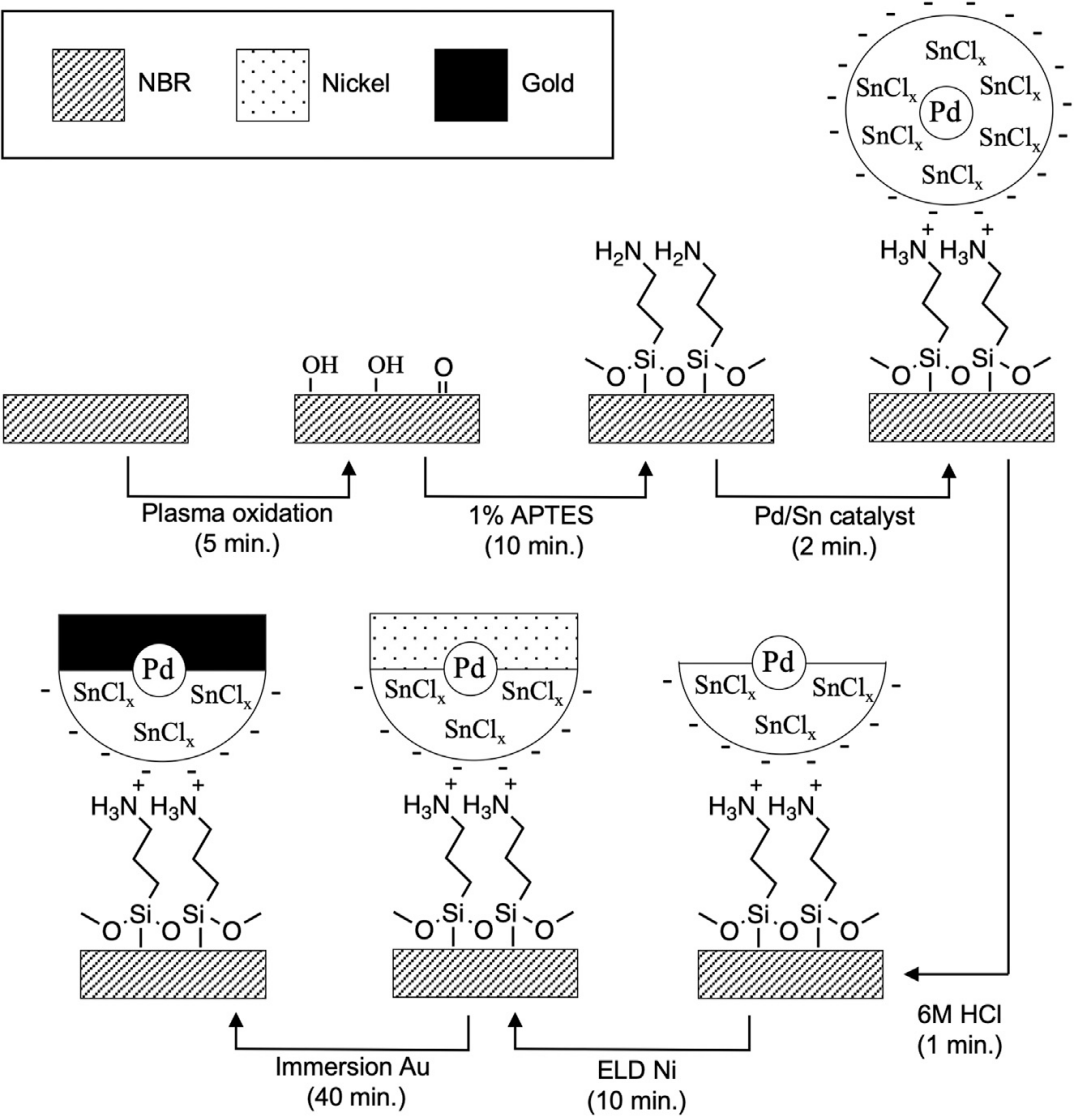
Fabrication of ENIG films on NBR Scheme adapted from Mechael et al. (2021).

The electrical properties and performance of the ENIG/NBR sensors rely on the quality of the gold coating, which is dictated by steps of the ENIG protocol that control film adhesion, thickness, uniformity, and composition. Therefore, a conceptual understanding of the ENIG procedure is vital for successful sensor fabrication and troubleshooting. Scheme 1 outlines the fabrication of ENIG films on NBR gloves. In the first step, plasma oxidation increases the wettability of the surface by generating hydrophilic functional groups. A subsequent series of solution-based reactions chemically modifies the surface to activate it for Ni ELD. Exposure to a solution of the organosilane adhesion promoter 3-aminopropyltriethoxysilane (APTES) results in chemisorption via a condensation reaction between hydrolyzed silanol groups of APTES and hydroxy groups on the surface of NBR, forming an amine-terminated NBR surface. Subsequent immersion in a solution of a palladium-tin (Pd/Sn) colloidal catalyst results in binding of the colloids to the modified NBR surface (Mechael et al., 2021). Pd/Sn colloids have a core-shell structure with a catalytic Pd core and a negatively charged tin chloride (SnCl_x_) shell. The acidic pH of the Pd/Sn colloidal suspension protonates the amine groups on the NBR surface to generate a positively charged ammonium surface that then binds the colloids through electrostatic interactions. Exposure of the catalyzed NBR surface to an accelerator solution of hydrochloric acid (HCl) partially etches the SnCl_x_ shell to expose the catalytic Pd core (Cohen and Meek, 1976). After immersion in the ELD Ni solution, the exposed Pd catalyzes the reduction of Ni^2+^ ions from solution to metallic Ni^0^ on the NBR surface (Krulik, 1982; Mallory and Hajdu, 1990). The Ni deposition then proceeds autocatalytically with the consumption of a reducing agent, dimethylamine borane (DMAB), in the plating solution (Mallory and Hajdu, 1990). The duration of immersion in the Ni ELD solution determines the thickness of the final Ni film. Finally, immersion in a gold cyanide salt solution results in the galvanic displacement of metallic Ni^0^ at the sample surface by Au^+^ in solution, generating a metallic film of Au^0^ on the surface while releasing Ni^2+^ ions into the solution (Accogli et al., 2020; Liu et al., 2007).

**Scheme 2:**
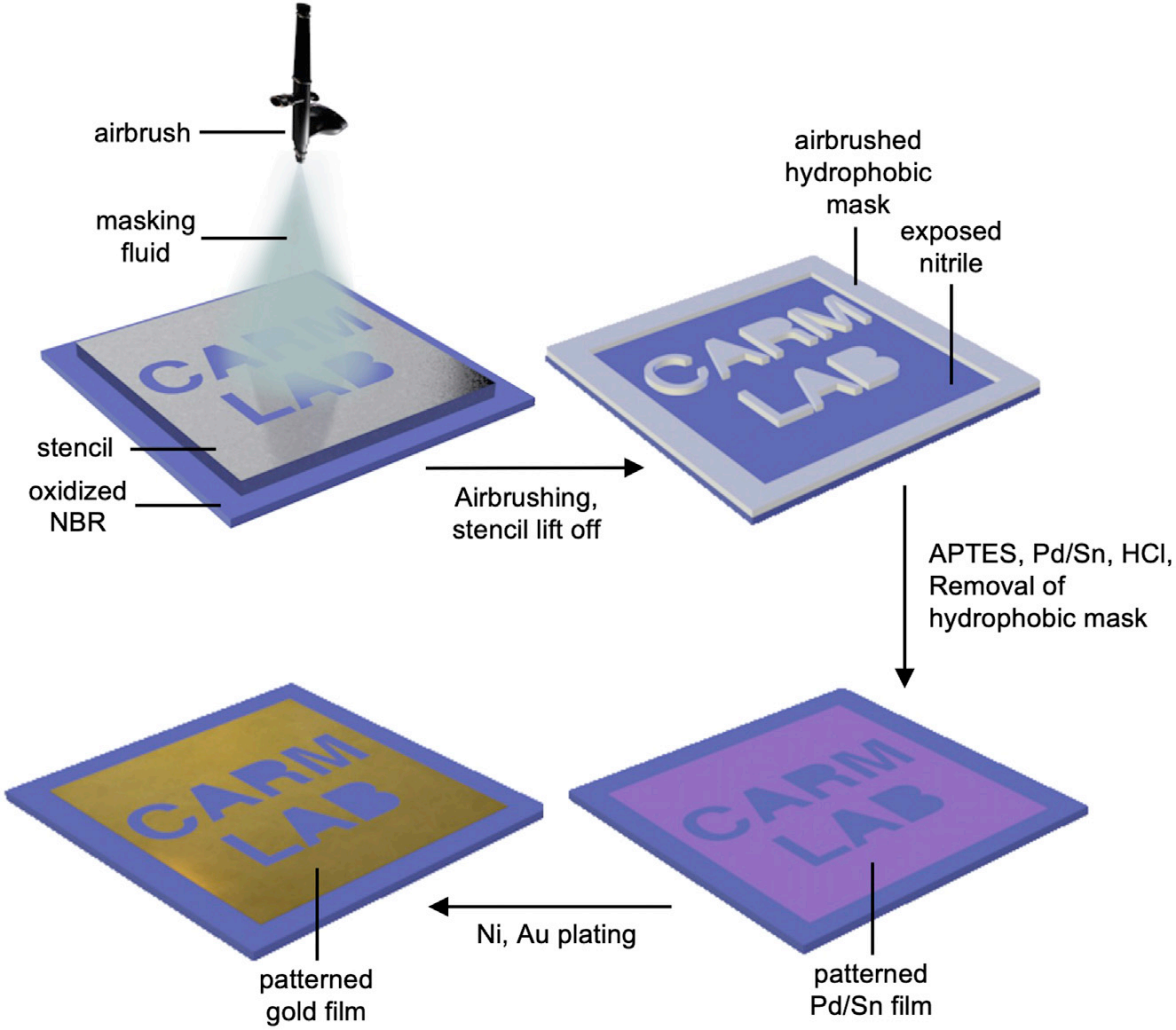
Fabrication of patterned ENIG films on NBR by spray coating a hydrophobic resist

Patterning of ENIG films on NBR to prepare the strain sensor array can be achieved by selectively blocking regions of the NBR surface from binding the Pd/Sn colloidal catalyst, thus preventing metal deposition in those regions. This protocol uses a spray-coating method in which airbrushing a commercial latex emulsion through a stencil mask generates a pattern of hydrophobic latex resist on the oxidized NBR surface. This latex emulsion is an art masking fluid that is available at art stores and is commonly applied on paper to block watercolor paints. This patterned latex resist on the NBR glove blocks the chemisorption of APTES and binding of Pd/Sn colloids (Scheme 2). Subsequently removing the latex mask reveals a visible tinted pattern on the surface due to the pattern of bound Pd/Sn colloids. Subsequent ELD Ni and immersion gold plating deposits the metallic coating only in the areas with bound Pd/Sn colloids, creating a patterned gold film.

Before you begin, you will need to prepare each plating solution using commercially available reagents. Because of the additive nature of this deposition technique and the stability of the Pd/Sn colloidal catalyst, HCl accelerator, and immersion gold solution, it is possible to reuse the solutions until they are either destabilized or depleted. Below, we describe the lifetime of these solutions and how to assess their stability.

### Preparing the Pd/Sn catalyst solution

**Timing: The solution preparation will take <10 min; however, the stability of the solution allows it to be reused until the colloids are destabilized (see** pause point **below).**

**Table:** 

Reagent	Final concentration	Amount
Cataposit 44 catalyst concentrate	3% v:v	3 mL
Cataprep 404 concentrate	0.27 g/mL	27 g
dH_2_O	n/a	97 mL
**Total**	**n/a**	**100 mL**

1. Weigh out 27.0 g of the Cataprep 404 concentrate in a 125 mL polypropylene (PP) bottle.
2. Add 85.0 mL of distilled water (dH_2_O) to the Cataprep 404 concentrate and shake to dissolve.
3. Add 3.0 mL of the Cataposit 44 catalyst concentrate and shake until color is uniform.
4. Add another 12.0 mL of dH_2_O to complete the volume and shake well to mix.

**Figure 1:**
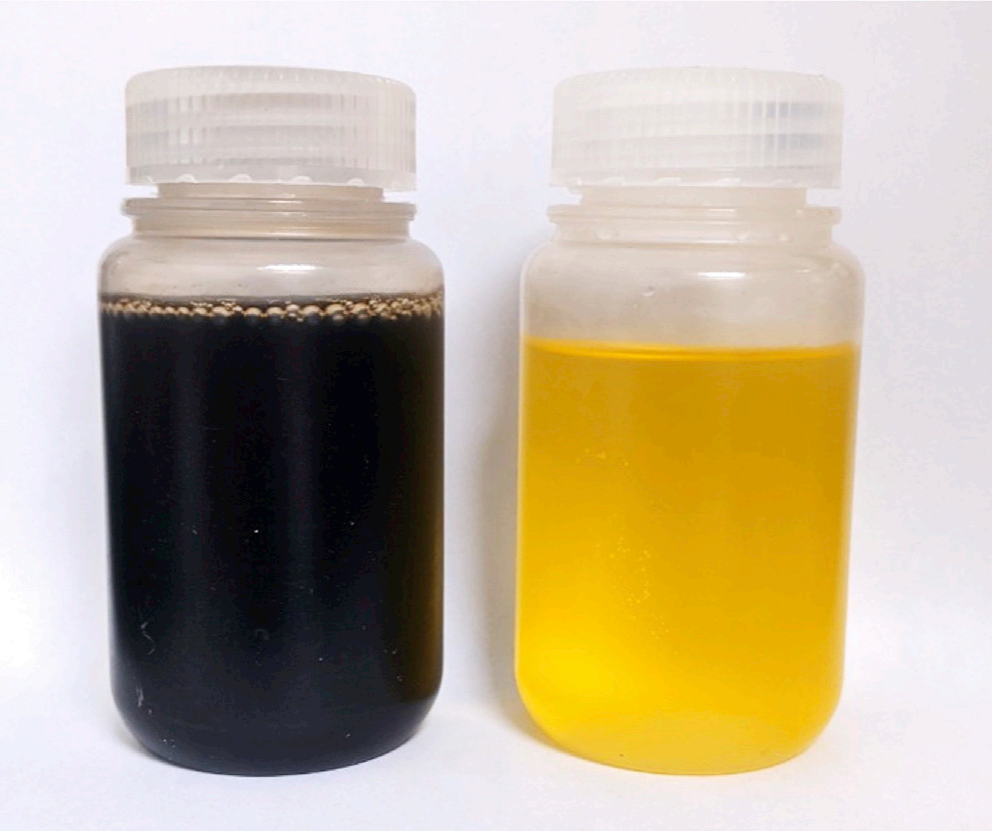
Photograph of a freshly prepared Pd/Sn solution (left) next to an aged solution (right)

**Pause point:** The final Pd/Sn solution can be stored at room temperature (20°C–25°C) and can be reused until the colloids are destabilized (lifetime depends on usage), which is visible by an abrupt colour transition from opaque dark brown to translucent yellow (Figure 1).

***Note:*** The Pd/Sn catalyst solution recipe reported here is based on the supplier’s instructions, which can be accessed here: https://ecems.net/tds/tdsdocs/Cataposit%2044.pdf

### Preparing the 6 M HCl accelerator solution

**Timing: The solution preparation will take <10 min; however, the stability of the solution allows it to be reused for several months.**

**Table:** 

Reagent	Final concentration	Amount
hydrochloric acid (HCl, 12 M)	6 M	50 mL
dH_2_O	n/a	50 mL
**Total**	**6 M**	**100 mL**

5. Add 50.0 mL of dH_2_O to a 125 mL PP bottle.
6. Measure out 50.0 mL of stock 12 M HCl and carefully add to the dH_2_O.

***Note:*** The final HCl accelerator solution can be stored at room temperature for 6 months.

### Preparing the electroless nickel (ELD Ni) solution

**Timing: The solution preparation will take <10 min and must be freshly prepared for each plating session.**

**Table:** 

Reagent	Final concentration	Amount
nickel (II) sulfate hexahydrate (NiSO_4_•6H_2_O)	0.08 M	2.1028 g
sodium pyrophosphate decahydrate (Na_4_P_2_O_7_•10H_2_O)	0.14 M	3.7226 g
dimethylamine borane (DMAB)	0.07 M	0.4250 g
dH_2_O	n/a	100 mL
**Total**	**n/a**	**100 mL**

7. Weigh out 3.7226 g of sodium pyrophosphate decahydrate and dissolve it in 100.0 mL of dH_2_O in a 125 mL PP bottle.
8. Weigh out 2.1028 g of nickel sulfate hexahydrate and add it to the PP bottle. Shake thoroughly to dissolve.
9. Weigh out 0.425 g of DMAB in a separate vial. Do not add this to the solution until you are ready to plate.

***Note:*** The final ELD Ni solution is not stable and should not be stored. To conserve the Ni in solution, it is best to add the reducing agent, DMAB, immediately before use.

### Preparing the immersion gold solution

**Timing: The solution preparation will take <15 min, however the stability of the solution allows it to be reused until all gold has been used up, which can take several months of regular use.**

**Table:** 

Reagent	Final concentration	Amount
Gobright TAM-55-R	6% v:v	30.0 mL
Gobright TAM-55-M10	10% v:v	50.0 mL
AURUNA 6700-Au Salts	n/a	1.0 g
dH_2_O	n/a	420 mL
**Total**	**n/a**	**500 mL**

**CRITICAL:** Review cyanide safety training before attempting to work with the AURUNA 6700-Au salts. The AURUNA 6700-Au salt contains cyanide, therefore all waste produced from the immersion gold solution must be kept separate from waste generated from the other plating solutions, which contain acids. Mixing cyanide with acids generates hydrogen cyanide (HCN) gas, which is lethal at exposures as low as 6.6 ppm depending on exposure duration (National Institute for Occupational Safety and Health (NIOSH), 2011).

10. Measure 20.0 mL of dH_2_O in a glass vial and heat to ∼ 80°C on a hot plate. Proceed with the next steps until you are ready to use the warmed dH_2_O (Step 15).
11. Measure 250.0 mL of dH_2_O and add it to a 1 L PP bottle.
12. Add 50.0 mL of Gobright TAM-55-M10 to the PP bottle and shake to mix.
13. Add 30.0 mL of Gobright TAM-55-R to the PP bottle and shake to mix.
14. Very carefully measure the AURUNA 6700-Au salt in a glass vial.
  a. Tare an empty capped glass vial on an analytical balance.
  b. Transfer AURUNA 6700-Au salt into a glass vial in a fumehood. Cap the vial in the fumehood before weighing the vial and Au salt on the analytical balance.
  c. Repeat until 1.0 g of AURUNA 6700-Au salt has been weighed out.
  d. Rinse anything that contacted the AURUNA 6700-Au salt with dH_2_O into the designated immersion gold waste bottle. All disposable materials that contacted the immersion gold solution should be disposed of in the designated cyanide solid waste.
15. Quantitatively transfer the AURUNA 6700-Au salt to the large PP bottle.
  a. Use a syringe to transfer 10 mL of the warmed dH_2_O from Step 10 to the AURUNA 6700-Au salt vial, ensuring no splashing occurs. Cap and swirl to dissolve the Au salt.
  b. Transfer the dissolved AURUNA 6700-Au salt to the large PP bottle. Swirl to mix.
  c. Use the same syringe to wash the empty Au salt vial with 5 mL of the warm dH_2_O. Transfer the wash to the large PP bottle. Repeat this step one more time.
16. Add 150.0 mL of dH_2_O into the large PP bottle. Swirl to mix.
17. Transfer the immersion gold solution to a 1 L jacketed beaker on a stir-plate that is attached to an oil circulation system (See materials and equipment). Cover the double-walled beaker when not in use.

**CRITICAL:** The immersion gold beakers must be covered when not in use and covered as much as possible during use to prevent evaporation of the solution.

**Figure 2:**
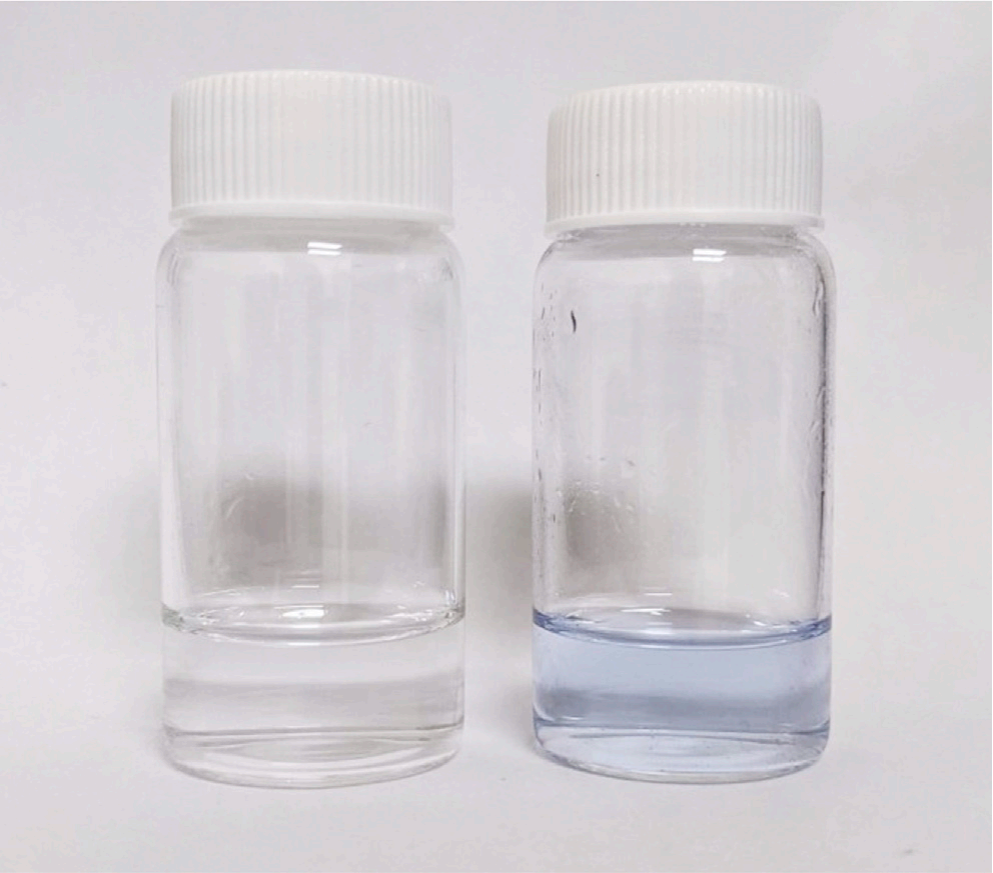
Photograph of a freshly prepared immersion gold solution (left) next to a depleted immersion gold solution (right)

***Note:*** The final immersion gold solution is reusable until the gold has been used up, which is visible by a slight blue-tinting of the solution (Figure 2).

***Note:*** To replace an immersion gold solution that has been depleted, use a 20 mL syringe to drain the depleted solution into the immersion gold waste bottle. Use a new 20 mL syringe to rinse the jacketed beaker with water three times, and then pour the freshly prepared immersion gold solution into the jacketed beaker. All syringes that contacted the immersion gold solution should be disposed of in the designated cyanide solid waste.

**Pause point:** The final immersion gold solution can be stored at room temperature in a closed container in a designated cyanide storage cabinet, or a covered beaker in a fume-hood.

## Key resources table

**Table:** 

REAGENT or RESOURCE	SOURCE	IDENTIFIER
**Chemicals, peptides, and recombinant proteins**		

3-Aminopropyltriethoxysilane (APTES)	Sigma-Aldrich	CAS 919-30-2
Cataposit 44 Catalyst Concentrate	Dow	N/A
Cataprep 404 Concentrate	Dow	N/A
hydrochloric acid (HCl)	Fisher Chemical	CAS 7647-01-0
nickel (II) sulfate hexahydrate	Oakwood Chemical	CAS 10101-97-0
sodium pyrophosphate decahydrate	Sigma-Aldrich	CAS 13472-36-1
dimethylamine borane (DMAB)	Oakwood Chemical	CAS 74-94-2
Gobright TAM-55 (TAM-55-R, TAM-55-M10, AURUNA 6700-Au Salts)	Uyemura	N/A
poly(octadecenyl-alt-maleic anhydride) (POMA)	Sigma-Aldrich	Cat#776866
Art Masking fluid	Winsor & Newton	N/A
Eutectic gallium-indium (EGaIn)	Sigma-Aldrich	Cat#495425

**Other**		

Polypropylene bottles (125 mL)	Fisher Scientific	Cat#02–896C
Polypropylene bottles (1.5 L)	Fisher Scientific	Cat#02–896F
Neo for Iwata CN gravity feed dual action airbrush	Iwata	Cat#N4500
Plasma cleaner and flow meter	Harrick Plasma	Cat#PDC-001, Cat#PDC-FMG
Bransonic ultrasonic cleaner	Sigma-Aldrich	Cat#Z244899
VWR Traceable platinum thermometer	VWR	Cat#36934-160
Huber ministate 125 circulator	Huber	N/A
Polypropylene scissor forceps	Thermo Scientific	Cat#6320-0010
Keithley sourcemeter 2601A	Keithley	N/A
Microvice stretcher	S.T. Japan	N/A
Chemglass 1000 mL jacketed reaction beaker	Chemglass	Cat#CG-1103-05
Slotted beaker cover	Made locally	N/A
VWR Traceable 3-line alarm timer	VWR	Cat#62344-912
Nitrogen spray gun	NCI	Cat#TA-N2-2000FT

## Materials and equipment

**Figure 3:**
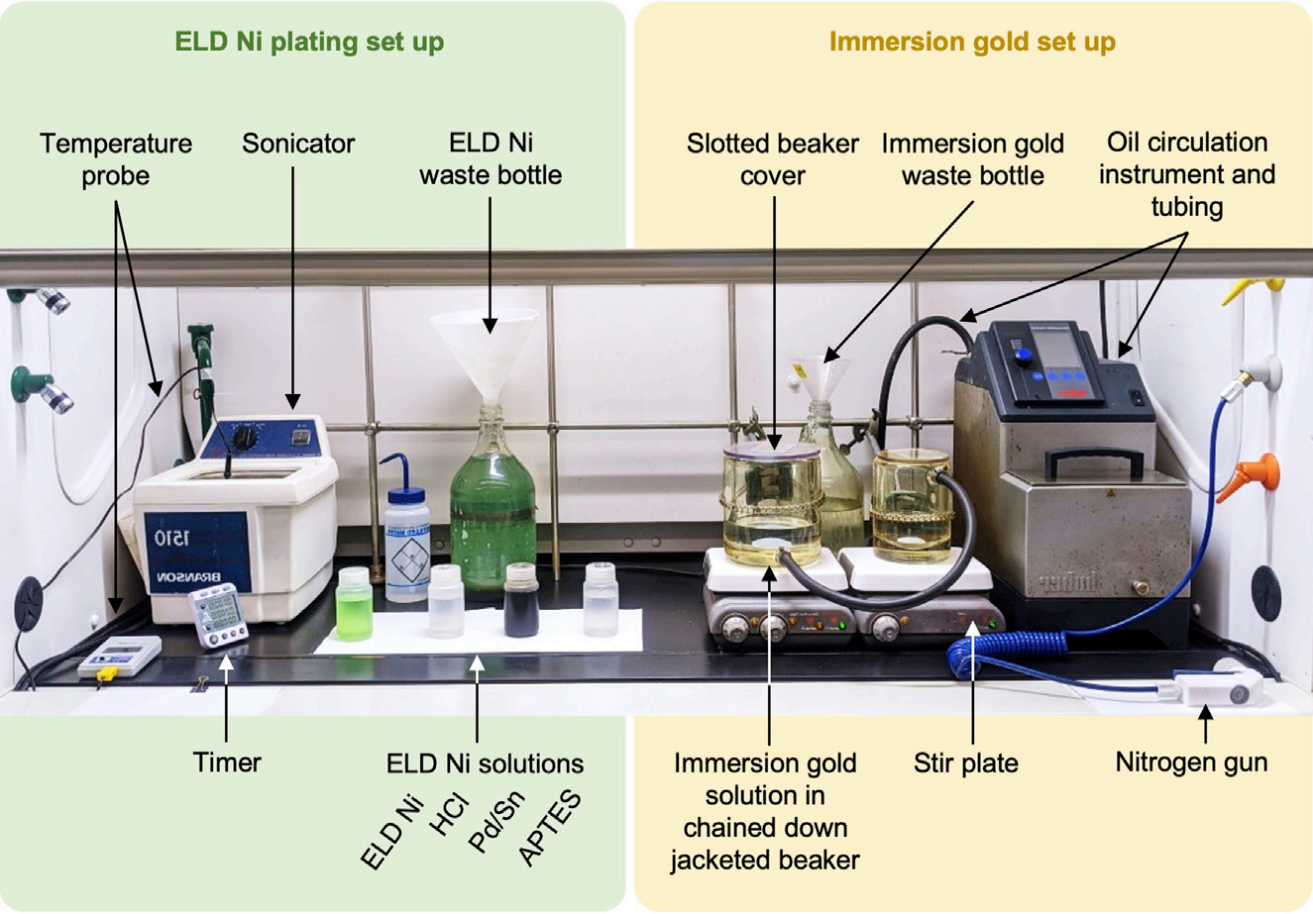
A fumehood equipped for ENIG deposition

The jacketed beakers containing immersion gold solution should be chained down to a mounted fumehood lattice frame to prevent spilling. The jacket of the beaker should be connected to a temperature-controlled oil circulation system to ensure quick and uniform temperature control of the immersion gold solution. Figure 3 shows a fumehood equipped with the equipment and solutions needed to carry out the ELD Ni and immersion gold steps of the protocol.

***Alternatives:*** Surface modification by APTES, Pd/Sn, HCl, and ELD Ni can alternatively be done in wide-mouthed containers such as plastic beakers or polystyrene petri dishes (except for ELD Ni).

## Step-by-step method details

### Masking patterns on the NBR surface

**Timing: 30 min**

The NBR surface must be activated to the ENIG process. The following steps describe the oxidation of the NBR surface by plasma oxidation to create a hydrophilic surface (water contact angle < 10 °), followed by a patterning method where a hydrophobic latex mask is airbrushed on the NBR surface. This mask will block the subsequent chemical modification of the NBR surface, effectively patterning the final metal deposition. Therefore, the airbrushing step is largely responsible for the final line-edge resolution of the deposited metal.

1. Oxidize the NBR samples in oxygen plasma (Harrick Plasma, PDC FMG) by placing the samples in the center of the chamber and oxidizing for 5 min at RF power of 10.2 W, a pressure of 1500 mTorr, and a flow rate of 63 mL/min at room temperature. If you suspect a problem with the oxidation of the NBR surface, see troubleshooting 1.

***Optional:*** It is useful to ensure the oxidation was successful by contact angle goniometry. The successful oxidation should give a water contact angle <10 °. It is recommended to do this once to confirm the oxidation conditions are sufficient, but it is not necessary to check the contact angle during each plating session.

2. If you are preparing patterns on small cut-out samples:
  a. Apply double sided tape to the surface of the NBR glove and use scissors to cut out NBR in the desired sample size.
  b. Laminate the sample to a glass slide gently pressing out air bubbles.
  c. Apply the stencil mask to the surface of the NBR using weak double-sided Scotch tape. Use as little tape as possible.
  d. Load the airbrush chamber with artists’ masking fluid. Holding the airbrush about 15 cm away and at a 90 ° angle to the sample, quickly airbrush a very thin film of artists’ masking fluid.
  e. Quickly remove the stencil mask and tape using tweezers taking care not to smear the masking fluid. Let the latex stand for 5 min or until completely dry.
  f. Clean the airbrush before the artists’ masking fluid dries.
3. If you are preparing patterns on a whole glove:
  a. Prepare a template glove by marking the locations where metal will be plated. If you will plate on specific locations on the hand (e.g., joints), you can put the glove on to get a better approximation of the correct location.
  b. Obtain another glove and turn it inside out. This will be the glove that you plate on.
  c. Overlay the template glove on the sample glove and use the template glove as a guide to fix a stencil mask in the desired plating locations using weak double-sided tape. Use as little tape as possible.
  d. Use small amounts of tape to tape the glove down to a flat surface such as a glass slide or benchtop. Ensure the glove is not wrinkling in the desired plating locations.
  e. Load the airbrush chamber with artists’ masking fluid. Holding the airbrush about 15 cm away and at a 90 ° angle to the sample, quickly airbrush a very thin film of artists’ masking fluid in the regions directly around the stencil masks.
  f. Quickly remove the stencil mask and tape using tweezers taking care not to smear the masking fluid. Let the latex stand for 5 min or until completely dry.
  g. Clean the airbrush before the artists’ masking fluid dries.
  h. Apply masking fluid to the remaining uncovered portions of the glove using a swab. Let the latex stand for 5 min or until completely dry.
  i. Compact the glove by folding in the palm side and fingers against a glass slide while keeping the desired plating regions exposed. While folding the glove, keep the open end of the glove exposed and leave space between the open end and the desired plating locations. The dried latex mask will adhere to itself, so no tape is needed to keep the glove compact. The final compacted glove should be small enough to fit in each ENIG plating solution.

**CRITICAL:** Spray coating generates aerosol latex drops. It is best to perform this step in a fumehood to avoid inhaling aerosols.

**Figure 4:**
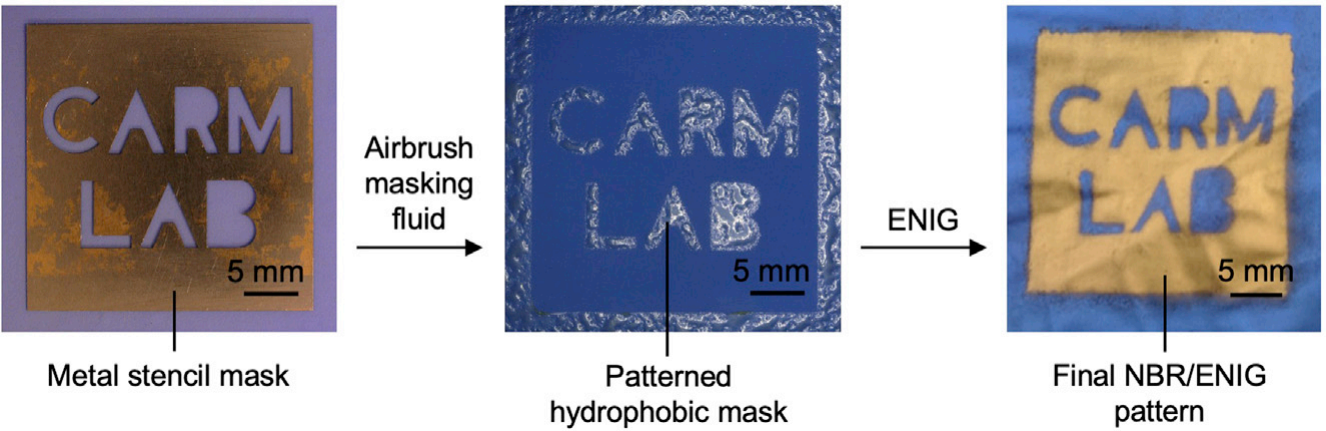
Photographs of NBR illustrating the patterning process

**CRITICAL:** The stencil mask used during airbrushing should be the “positive” pattern that you want to metallize. The stencil mask is being used to prepare the hydrophobic latex mask, which will be the “negative” pattern that will be metallized during the ENIG process. An example is shown in Figure 4.

**CRITICAL:** The open end of the glove must be kept exposed. During the plating process, the open end of the glove will not be immersed in the solution to avoid chemical exposure to the inside of the glove.

***Note:*** The artists’ masking fluid dries quickly and can clog the airbrush. Be sure to quickly clean the airbrush before the masking fluid dries. To do this, quickly pour out excess masking fluid from the paint chamber and rinse the chamber with water. Then fill the chamber with water and airbrush the water out. Refill and repeat the water rinse three times. If the trigger of the airbrush begins sticking, take apart the airbrush and remove dried latex that may have accumulated around the needle or trigger.

***Note:*** When fixing the stencil mask to the NBR sample using double sided tape, ensure adhesive from the double-sided tape does not transfer to the NBR. Any remaining adhesive will block the ENIG deposition. To avoid this, use as little tape as possible, use weak double-sided tape, and/or weaken the adhesive by laminating the tape repeatedly on another surface.

### Electroless nickel immersion gold plating

**Timing: ∼1.5 h**

The following steps describe the sequential chemical modification of the NBR surface for ENIG deposition. This first involves amine functionalization by 3-aminopropyltriethoxysilane (APTES) that is subsequently protonated to electrostatically bind the Pd/Sn catalytic colloids. The Pd core is then exposed by etching the SnCl_x_ shell in the HCl accelerator solution. The ELD Ni solution will deposit a Ni film on the NBR surface. This step is largely responsible for the film thickness and can be adjusted by adjusting the Ni plating time. Finally, immersion in the immersion gold solution results in the galvanic displacement of nickel with gold ions from the solution to obtain a gold film. The immersion duration in the gold solution can be done in excess to ensure complete replacement of nickel with gold (< 5 wt% Ni in the final film).

4. Prepare a 1% (v/v) solution of APTES in dH_2_O in a 125 mL PP bottle.
5. Submerge the NBR samples in the 1% APTES solution for 10 min. If you suspect a problem with the surface functionalization by APTES, see troubleshooting 2. Rinse the sample in water and dry the surface with a stream of nitrogen.

***Optional:*** If you are using a Huber oil circulation system, perform an air-purge for 15 min. Then heat the oil to 60°C while stirring the gold solution using a magnetic stir bar. Proceed with the ENIG procedure while the solution heats.

6. Add the pre-weighed DMAB reducing agent to the ELD Ni solution and sonicate the Ni solution while maintaining a water temperature of ∼30°C in the sonicator for the duration of the plating session.

**Figure 5:**
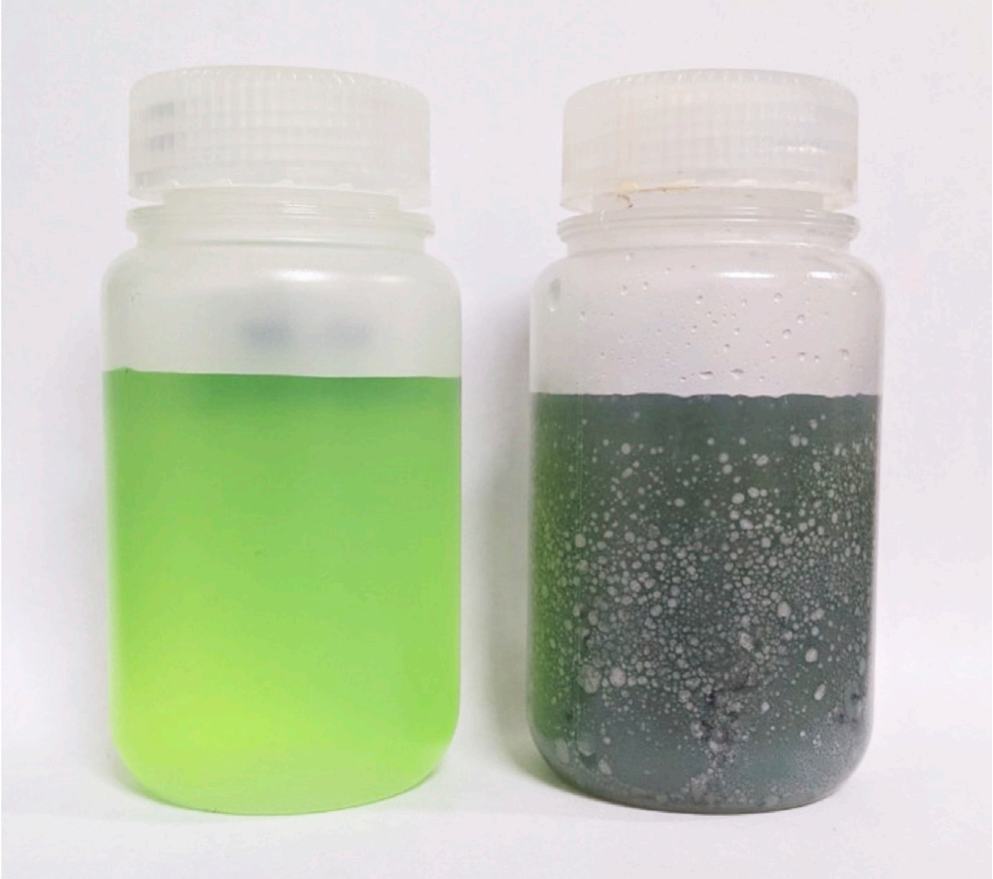
Photograph of a freshly prepared ELD Ni solution (left) next to a solution that has plated out (right)

**CRITICAL:** After the addition of the DMAB reducing agent, the ELD Ni solution has a useful plating rate for about 2 h, after which the Ni solution becomes unstable and will “plate out,” meaning metallic Ni will begin to rapidly precipitate from solution (Figure 5).

7. Submerge the NBR sample into the Pd/Sn solution for 2 min. Thoroughly rinse the sample with water and dry with a stream of nitrogen. You should see a slight purple-brown tint on the desired plating locations. If you do not see this and you do not get uniform Ni deposition after step 10, see troubleshooting 3.
8. Submerge the NBR sample into the 6 M HCl accelerator solution for 1 min. Thoroughly rinse the sample with water and dry with a stream of nitrogen.
9. Peel away the hydrophobic latex mask with tweezers. Light friction may be applied to create a delamination point. Ensure no friction is applied on the desired plating locations. Remove any tape from the sample and clip the sample flat to a glass slide using scissor forceps.
10. Submerge the NBR sample in the ELD Ni solution for 10 min with sonication. Thoroughly rinse the sample with water and dry with a stream of nitrogen. If you do not obtain uniform Ni deposition, see troubleshooting 4. If you see metal deposition in undesired locations, see troubleshooting 5.

***Note:*** Adhesives from tapes can compromise the ELD Ni solution. It is best to use non-metallic clips such as polypropylene scissor forceps (see materials and equipment) to keep the sample flat against a glass slide in the ELD Ni solution.

11. Submerge the NBR sample in the heated immersion gold solution with very gentle stirring for 40 min. Ensure the stir bar is not hitting the sample. Thoroughly rinse the sample with water and dry with a stream of nitrogen. If you do not obtain uniform gold deposition, see troubleshooting 6. If the final ENIG/NBR sensor does not meet the expected outcomes, see troubleshooting 7, 8, 9, and 10.

***Optional:*** Adhesion of the ENIG film to the NBR substrate can be assessed by performing a tape test, which involves laminating scotch tape to the surface of the ENIG coating and then peeling the tape away. If the tape removes portions of the ENIG coating, then this indicates a problem in the surface activation steps of the protocol (see troubleshooting 1, 2, 3 and 7 to ensure the surface activation was successful).

## Expected outcomes

**Figure 6:**
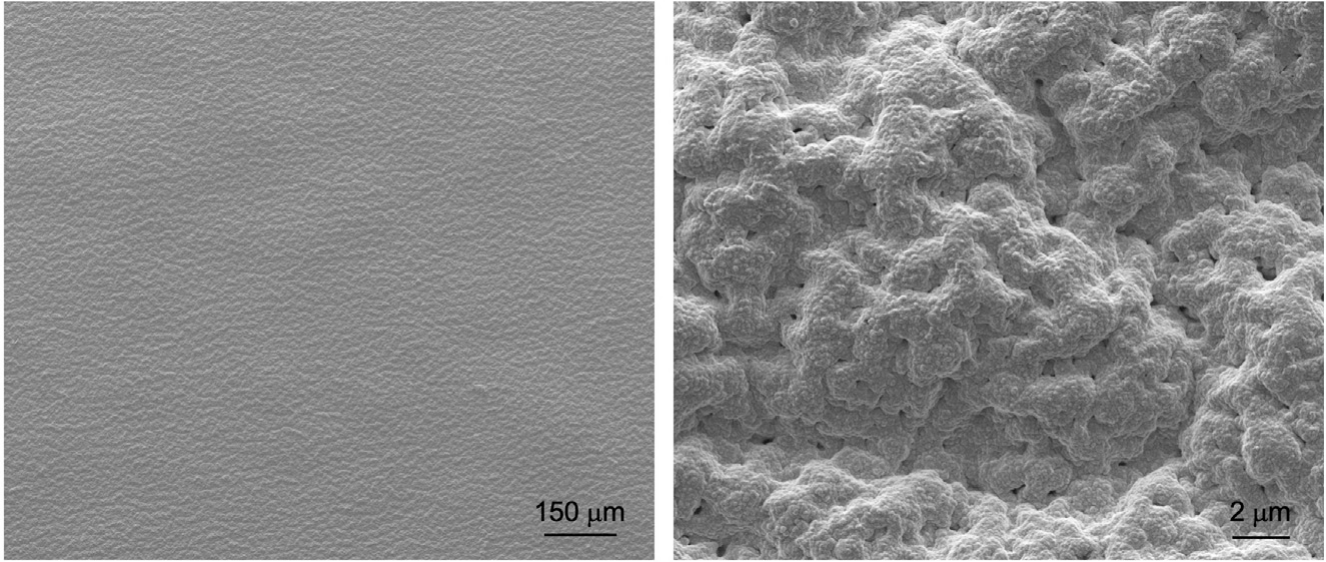
Scanning electron microscope images of the ENIG/NBR surface

**Visual and compositional outcomes:** The resulting ENIG coating on NBR should be defect-free and conformal (Figure 6).

**Figure 7:**
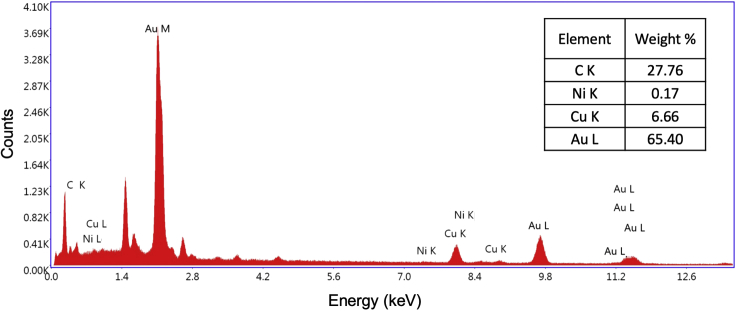
Energy-dispersive x-ray spectroscopy (EDS) analysis of the ENIG/NBR surface Figure adapted from Mechael et al. (2021).

The ENIG/NBR sensor will have < 5 wt% Ni. An example of the elemental composition characterized by energy-dispersive x-ray spectroscopy (EDS) is displayed in Figure 7. If you do not visually or spectroscopically observe displacement of Ni with Au after the immersion gold step, see troubleshooting 6.

**Expected electrical metrics:** The sheet resistance of the ENIG/NBR sensors is 3.1 ± 0.6 Ω/sq and a sensor with dimensions 2 cm **×** 0.5 cm will have an initial resistance of 8 ± 3 Ω over the 2 cm length. The electrical resistance is relatively stable and should not exhibit large changes with age. If the resistance of the sensors shows signs of instability, see troubleshooting 9.

**Figure 8:**
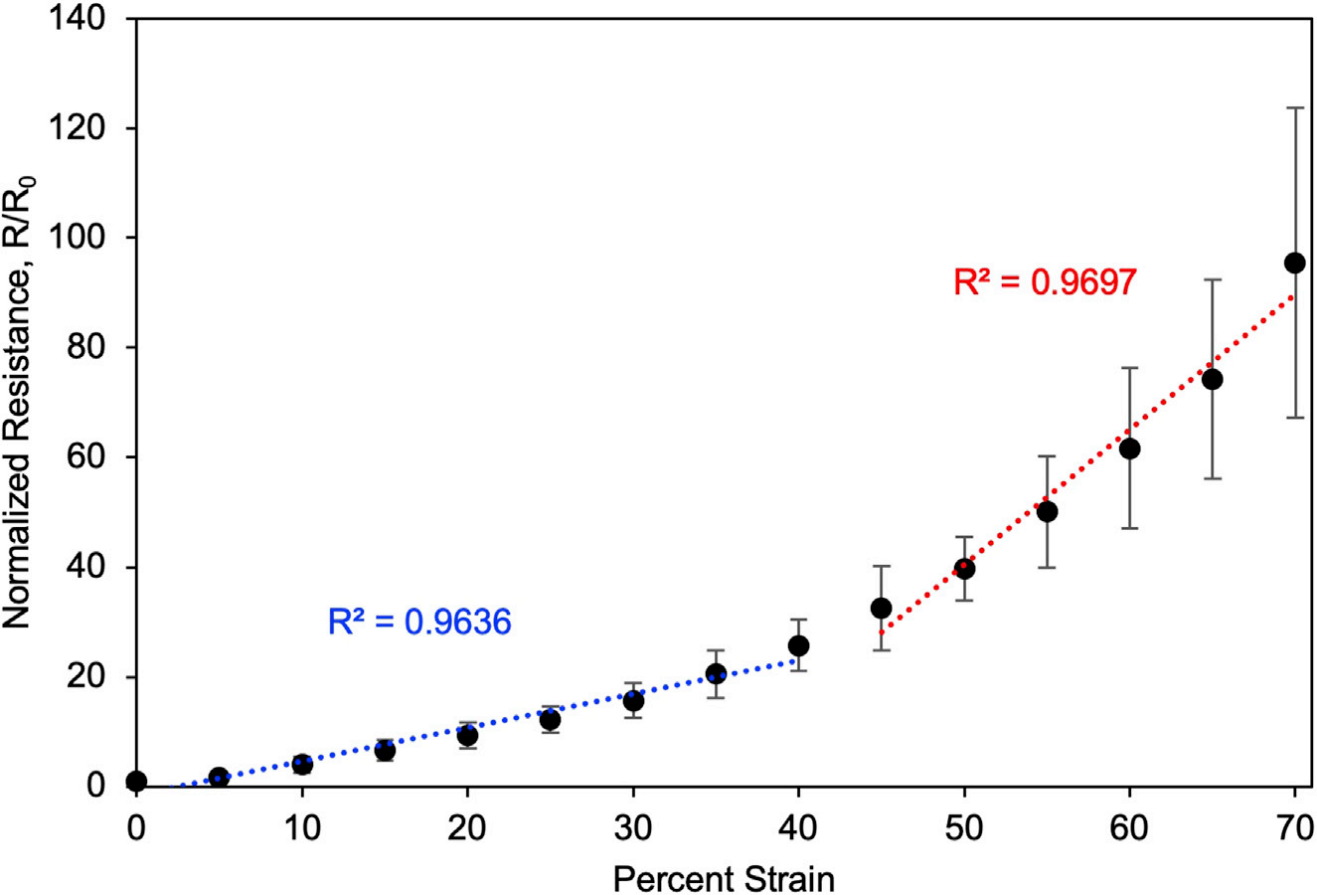
Normalized change in resistance (R/R_0_) as a function of percent strain of the ENIG/NBR sensors Data points represent mean ± standard deviation. Plot adapted from Mechael et al. (2021).

**Expected working range:** The resistance of a 2 cm **×** 0.5 cm sensor increases to ∼85**×** the initial resistance at 70% strain. Above 70% strain, the sensors may experience electrical failure. Figure 8 shows the expected strain response of a 2 cm **×** 0.5 cm ENIG/NBR sensor. The resistive strain response of the sensor can be fitted by two linear regions from 0%–40% strain, and 45%–70% strain with correlation coefficients (R^2^) > 0.96. If the sensors exhibit electrical failure at low strains, see troubleshooting 10.

**Expected strain sensitivity:** The sensitivity of the ENIG/NBR sensor is quantified by calculating the gauge factor (GF) over each linear working range. GF is calculated as the change in resistance over a defined strain range, where R is resistance, R_0_ is initial resistance, and ε is the mechanical strain:

$$\text{GF}=\left(\left({\text{R}-\text{R}}_{\text{0}}\right)/{\text{R}}_{\text{0}}\right)/\text{ε}$$

The ENIG/NBR strain sensors demonstrate high sensitivity with a GF of 62 between 0%–40%, and a GF of 246 between 45%–70% strain.

## Quantification and statistical analysis

The following is a description of the electromechanical data processing, statistical analysis, and criteria for data exclusion. Analysis of electromechanical data should be based on the performance of at least three sensors, however, for best practice, characterizing more sensors is encouraged to confidently represent the behavior. Table 1 shows an example of data processing and statistical analysis for three ENIG/NBR sensor samples.

1. Clamp the ENIG/NBR sensors in a micro-vice stretcher (S.T. Japan, USA, Inc).
2. Measure resistance, R, during stretching at 5% increments of the sample’s initial length using a Keithley 2601A Sourcemeter. Measure the resistance until electrical failure of the sensor is observed, as reflected by a GΩ resistance measurement by the sourcemeter.
3. Calculate normalized resistance, R/R_0_, to describe how the resistance has changed relative to that sensor’s initial resistance, which minimizes variation in the response deriving from initial resistance and clamp-to-clamp distance variations. The electromechanical behavior is reported as the mean R/R_0_ ± standard deviation.

***Note:*** A sample may be excluded if you have reason to believe the ENIG protocol was not successful (e.g., non-uniform metal deposition) or if the sample was damaged/stretched during handling since this will impact subsequent cracking in the ENIG film.

**Figure 9:**
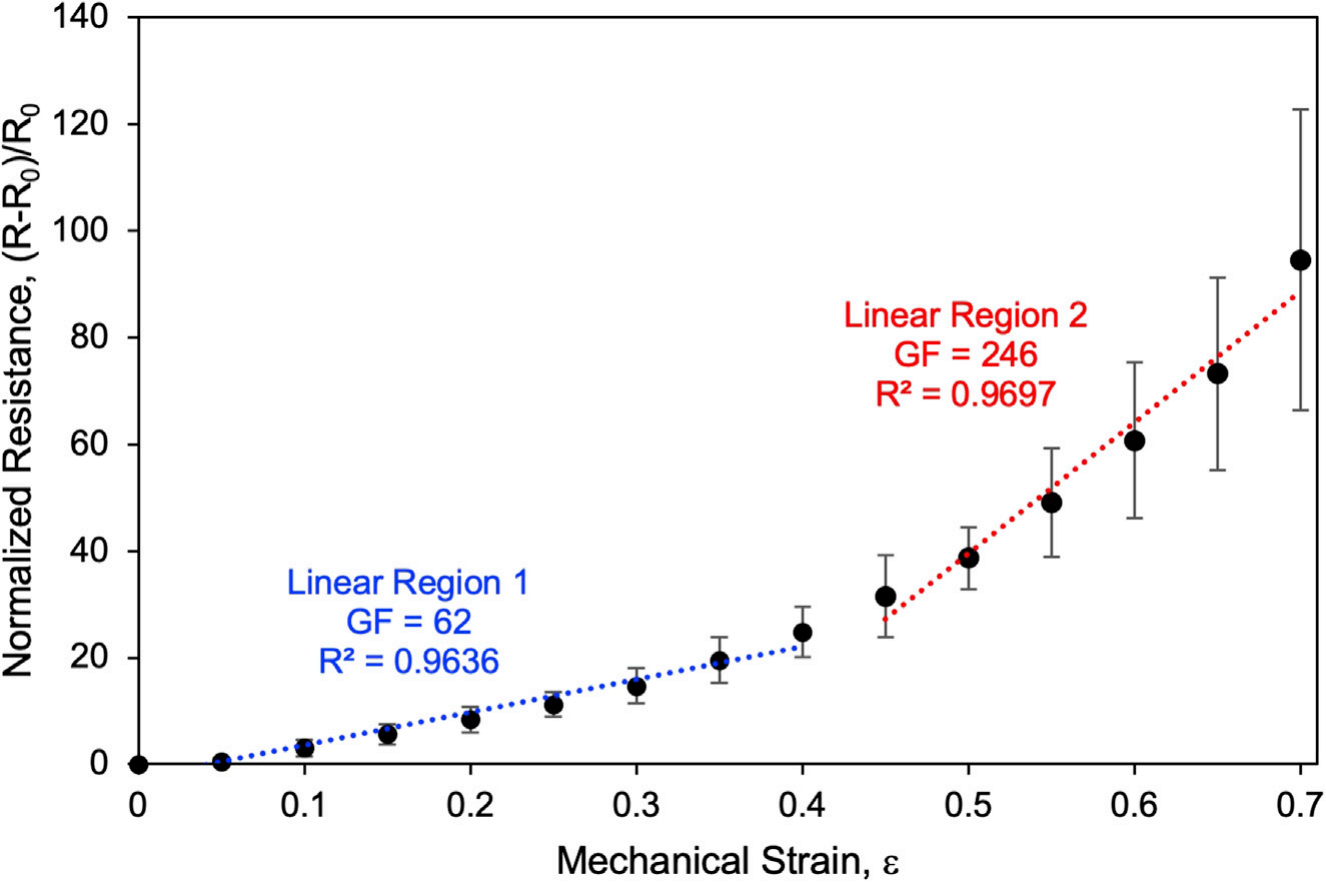
Normalized change in resistance ((R-R_0_)/R_0_) as a function of mechanical strain of the ENIG/NBR sensors Data points represent mean ± standard deviation. Slope represents gauge factor (GF). Plot adapted from Mechael et al. (2021).

4. Select the linear fits of the resistance change during strain to achieve the highest possible correlation coefficients.
  a. For the dataset provided in Table 1, two linear regions were selected to describe the sensing behavior between 0%–40% and 45%–70% strain.
  b. Table 2 shows an example of how the raw resistance data is processed to calculate GF for the two linear regions. The resistance measured by the sourcemeter is normalized as (R-R_0_)/R_0_, which is the numerator of the GF equation. The mean normalized resistances are used to calculate GF. Since we are using two linear fits to describe the behavior of the ENIG/NBR sensors, we calculate gauge factor for each of the linear regions as the slope of the normalized resistance over the linear region (Figure 9).

**Table 2:** Example of sensitivity data processing

Ε	Sample 1		Sample 2		Sample 3		Average	
	R (Ω)	(R-R_0_)/R_0_	R (Ω)	(R-R_0_)/R_0_	R (Ω)	(R-R_0_)/R_0_	(R-R_0_)/R_0_	GF
**0.00**	11.2	0.00	7.07	0.00	5.35	0.00	0.00	62
**0.05**	20	0.79	13.9	0.97	5.45	0.02	0.59	
**0.10**	52.9	3.72	35.36	4.00	12.44	1.33	3.02	
**0.15**	88.5	6.90	53.18	6.52	23.94	3.47	5.63	
**0.20**	127	10.34	71.57	9.12	35.71	5.67	8.38	
**0.25**	154	12.75	94.59	12.38	51.17	8.56	11.23	
**0.30**	202	17.04	121	16.11	64.54	11.06	14.74	
**0.35**	265	22.66	159	21.49	83.2	14.55	19.57	
**0.40**	334	28.82	191	26.02	110	19.56	24.80	
**0.45**	446	38.82	236	32.38	131	23.49	31.56	246
**0.50**	498	43.46	293	40.44	178	32.27	38.73	
**0.55**	675	59.27	355	49.21	213	38.81	49.10	
**0.60**	864	76.14	422	58.69	258	47.22	60.69	
**0.65**	1023	90.34	537	74.95	296	54.33	73.21	
**0.70**	1381	122.30	681	95.32	358	65.92	94.51	
**0.75**	1847	163.91	829	116.26	Failure			
**0.80**	2262	200.96	1309	184.15				
**0.85**	2974	264.54	1419	199.71				
**0.90**	3816	339.71	2018	284.43				
**0.95**	5931	528.55	3075	433.94				
**1.00**	8654	771.68	Failure					
**1.05**	13080	1166.86						
**1.10**	Failure

**Table 1:** Example of electromechanical data processing

% strain	Sample 1		Sample 2		Sample 3		Average	
	R (Ω)	R/R_0_	R (Ω)	R/R_0_	R (Ω)	R/R_0_	R/R_0_	Stdev.
**0**	11.2	1.00	7.07	1.00	5.35	1.00	1.00	0.00
**5**	20	1.79	13.9	1.97	5.45	1.02	1.59	0.50
**10**	52.9	4.72	35.36	5.00	12.44	2.33	4.02	1.47
**15**	88.5	7.90	53.18	7.52	23.94	4.47	6.63	1.88
**20**	127	11.34	71.57	10.12	35.71	6.67	9.38	2.42
**25**	154	13.75	94.59	13.38	51.17	9.56	12.23	2.32
**30**	202	18.04	121	17.11	64.54	12.06	15.74	3.22
**35**	265	23.66	159	22.49	83.2	15.55	20.57	4.38
**40**	334	29.82	191	27.02	110	20.56	25.80	4.75
**45**	446	39.82	236	33.38	131	24.49	32.56	7.70
**50**	498	44.46	293	41.44	178	33.27	39.73	5.79
**55**	675	60.27	355	50.21	213	39.81	50.10	10.23
**60**	864	77.14	422	59.69	258	48.22	61.69	14.56
**65**	1023	91.34	537	75.95	296	55.33	74.21	18.07
**70**	1381	123.30	681	96.32	358	66.92	95.51	28.20
**75**	1847	164.91	829	117.26	Failure			
**80**	2262	201.96	1309	185.15				
**85**	2974	265.54	1419	200.71				
**90**	3816	340.71	2018	285.43				
**95**	5931	529.55	3075	434.94				
**100**	8654	772.68	Failure					
**105**	13080	1167.86						
**110**	Failure							

## Limitations

The ENIG protocol described here is a sequence of surface modification steps, therefore, it is critical that the plasma activation of the substrate is successful. Although we have previously demonstrated ENIG on NBR, PDMS, polyester, and polyurethane, the surface activation by plasma oxidation can be a problem for other substrates where scission products are formed during oxidation (Vohra et al., 2016). It is important to confirm the successful activation of the substrate using contact angle or spectroscopic techniques (See troubleshooting 1 and 2). Some substrates and materials being immersed in the solutions may be incompatible with the 6M HCl accelerator solution. If this is a problem, an aqueous NaOH accelerator solution may be used instead.

## Troubleshooting

Here we will address problems you may encounter during ENIG deposition. We advise troubleshooting with an NBR sample as well as a control substrate such as clean glass. Assessing differences in the plating, such as successful deposition on the control substrate but failed deposition on the target substrate, can be an indication of a substrate problem such as unsuccessful oxidation.

### Problem 1

Confirming the oxidation of NBR surface using contact angle goniometry.

### Potential solution

The native NBR surface is hydrophilic and has a sessile water contact angle of 30 ± 11 °. Successful oxidation after step 1 of the protocol should decrease the contact angle to < 10 °. The effectiveness of plasma oxidation varies with pressure and flow rate, therefore, adjusting these variables using the manufacturer’s recommended settings may aid in successfully oxidizing the NBR surface.

### Problem 2

Confirmation of APTES modification using attenuated total reflection Fourier transform infrared (ATR-FTIR) spectroscopic detection of a POMA tag.

### Potential solution

**Figure 10:**
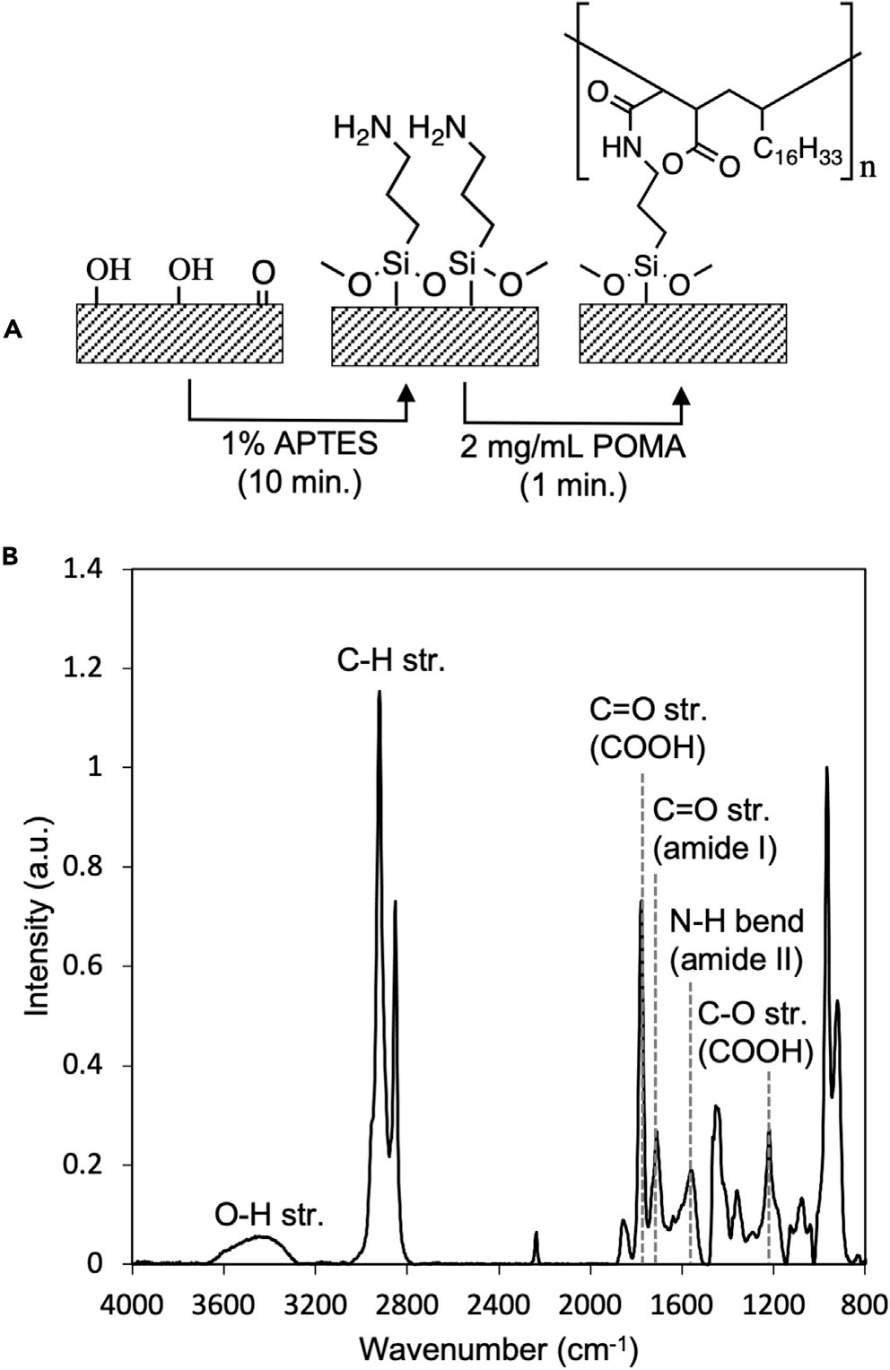
Verification of APTES modification by ATR-FTIR-detection of POMA tag (A) Scheme depicting the reaction of oxidized NBR with 1% APTES followed by labeling the APTES with POMA for IR detection. (B) ATR FT-IR spectra of NBR after APTES and POMA treatment. Figure adapted from Mechael et al. (2021).

Surface activation by APTES in step 5 of the protocol generates a primary amine surface which is challenging to detect spectroscopically. If you wish to confirm the surface modification by APTES, you can tag APTES with poly(octadecenyl-alt-maleic anhydride) (POMA) to generate IR-detectable amide and carboxylic acid stretches. To tag the APTES surface, dropcast a 2 mg/mL POMA solution in acetone on the sample surface and leave the solution for 1 min. Rinse the sample with acetone and sonicate in acetone for 10 min to remove physisorbed POMA. ATR-FTIR analysis will reveal the amide I stretch (1714 cm^−1^), the amide II stretch (1573 cm^−1^), and carboxylic acid stretches (1779 cm^−1^ and 1222 cm^−1^), consistent with the presence of POMA (Figure 10). If APTES cannot be detected by tagging with POMA, ensure you are successfully oxidizing the NBR surface (see troubleshooting 1).

### Problem 3

The sample has no visible tinting after immersion in the Pd/Sn solution.

### Potential solution

The presence of the Pd/Sn colloids is visible by a slight purple-brown tint in the desired plating regions. If you do not observe this tinting after step 7, it may still be revealing to move on and try ELD Ni plating since the Ni film is more visible and uniformity will be easier to assess. If you also do not observe Ni plating, it is likely that the Pd/Sn colloids have not bound to the desired plating regions during immersion in the Pd/Sn solution, which can be caused by problems in any of the prior steps (activation of NBR surface) or immersion in the Pd/Sn solution. First, confirm the activation of the NBR surface was successful (see troubleshooting 1 and 2). If the surface activation was successful, then the immersion time in the Pd/Sn solution was not long enough to electrostatically bind a uniform layer of the Pd/Sn colloids on the surface. Try increasing the immersion time in the Pd/Sn solution in 30 s increments.

### Problem 4

A nickel film did not form in the ELD Ni solution.

### Potential solution

If no nickel film is observed after completing step 10, first ensure DMAB was added to the ELD Ni solution and that it has had sufficient time to mix with the solution. You should leave the solution to sonicate for 5+ min prior to plating. If this often occurs with the first sample of a plating session, it is an indication that there is insufficient mixing time. Ensure the temperature of the sonication solution is being maintained at ∼ 30°C. Lower temperatures will result in slower Ni film deposition. Check that the pH of the ELD Ni solution is between 8–12 (Chen et al., 2019; Shi and Walker, 2011).

Next, confirm the presence of Pd/Sn on the NBR surface after immersion in the Pd/Sn solution (see troubleshooting 3). If the Pd/Sn tinting is visible after immersion in the Pd/Sn solution but is no longer visible after immersion in the HCl accelerator solution, then this may indicate the Pd/Sn colloids are being removed in the HCl solution prior to ELD Ni plating. The timing of the Pd/Sn and HCl immersion steps are critical to balance the exposure of the Pd catalytic core while maintaining a partially etched Sn shell to preserve electrostatic binding to the NBR surface. Long immersion times in HCl may completely etch the Sn shell, which in turn will release the catalytic Pd core from the surface. In contrast, short immersion times in HCl will not etch enough of the Sn shell and there will not be enough Pd exposed to catalyze the ELD Ni plating. To troubleshoot the immersion time of the HCl solution, start with the time reported in this protocol, and then increase or decrease the immersion time by 10–30 s increments.

### Problem 5

Nickel is plating in undesired, masked off regions.

### Potential solution

Very thin airbrushed layers of the artists’ masking fluid may not uniformly mask the NBR from plating. For the best patterning results in step 10, airbrush thick layers of masking fluid in step 2 or 3, taking care to spray evenly to avoid having the masking fluid accumulate and run on the surface.

### Problem 6

A gold film did not form during immersion in the gold solution.

### Potential solution

If gold is not displacing nickel on the surface in step 11, this is likely related to the immersion gold step or the thickness of the nickel film. The displacement of nickel by gold in the immersion gold solution is slow and requires heat and stirring. If you complete the immersion time reported in this protocol and do not observe gold deposition, try increasing the immersion time by 15–20 min increments. If you are not using a heated oil-circulation system, ensure the solution is being uniformly heated to ∼60°C. If the immersion gold solution is not being stirred, expect longer immersion times. If you are using a magnetic stir bar, ensure the stirring is set to the slowest rotation setting and is not hitting the sample.

Very thin nickel films appear matte and dark. These films may remain dark and matte or may be removed from the sample surface in the immersion gold solution. If this is the case, try increasing the ELD Ni plating time by 2 min increments and check the adhesion of the films by doing a tape test. To perform a quick tape test, laminate tape on the film and peel the tape off, checking if it removed any of the film from the surface. If the tape peels away the film from the NBR substrate, then there is poor adhesion (see troubleshooting 1, 2, and 3).

### Problem 7

The ENIG film is delaminating from the NBR substrate.

### Potential solution

Delamination of the ENIG film from the NBR substrate, especially after deformation of the sensor, is a result of poor adhesion. Delamination can be observed using an optical microscope and appears as flaking of the ENIG film at crack edges under strain. Delamination can also cause poor electrical recovery of the sensor after a single stretching cycle. Adhesion of the final ENIG film obtained after step 11 can be assessed by performing a tape test, which is described in the optional step following step 11. If the tape removes portions of the ENIG coating, then this indicates a problem in the surface activation steps of the protocol (see troubleshooting 1, 2, and 3 to ensure the surface activation was successful).

### Problem 8

The final ENIG films have high resistance or sheet resistance.

### Potential solution

The resistance and sheet resistance of the final ENIG/NBR sensor obtained after step 11 is largely dictated by the ENIG film thickness and quality. Increasing the thickness of the metal can lower the resistance and can be achieved by increasing the immersion time in the ELD Ni solution; however, Ni films that are too thick can result in blistering of the Ni film due to hydrogen gas production during the reduction of Ni by DMAB, leading to patches with poor adhesion. It is also important to keep the sample flat to avoid film damage from the sample curling/bending/folding during the ENIG procedure. Additionally, high resistances may be caused by an incomplete displacement of nickel by gold, which can be resolved by increasing the immersion gold plating time to ensure there is < 5 wt% Ni in the final ENIG film.

### Problem 9

The final ENIG films have a resistance or sheet resistance that changes significantly over time.

### Potential solution

We have seen a slight increase in resistance of the ENIG/NBR sensors obtained after step 11 from 3.1 ± 0.6 Ω/sq to 6.2 ± 0.3 Ω/sq over a 7-month period. Observing a significant change in the resistance over time can be caused by an incomplete displacement of Ni by Au during immersion gold deposition. To resolve this, increase the immersion gold plating time and ensure there is < 5 wt% Ni in the final ENIG film.

### Problem 10

The final ENIG/NBR sensors are experiencing electrical failure at low strains.

### Potential solution

The working range of the ENIG/NBR sensors obtained after step 11 can be influenced by the thickness, quality, and adhesion of the ENIG film. It is important to avoid introducing cracks and defects to the metal film during plating that may arise from physical handling, which can be minimized by keeping the sample flat against a platform such as a glass slide. Blistering during ELD Ni plating is another source of defects in the final ENIG film, caused by entrapment of hydrogen gas, which is a by-product of the reduction of Ni by DMAB. It is important to have sonication on during ELD Ni to remove hydrogen gas bubbles that may be pinned at the NBR surface. Another cause of poor electrical performance with strain is poor adhesion of the ENIG coating to the NBR substrate. Adhesion of the final ENIG film obtained after step 11 can be assessed by performing a tape test, which is described in the optional step following step 11. If the tape removes portions of the ENIG coating, then this indicates a problem in the surface activation steps of the protocol (see troubleshooting 1, 2, and 3 to ensure the surface activation was successful).

## Resource availability

### Lead contact

Further information and requests for resources and reagents should be directed to and will be fulfilled by the lead contact, Dr. Tricia Breen Carmichael (tbcarmic@uwindsor.ca).

### Materials availability

This study did not generate new unique reagents.

## Data Availability

The published article includes all data sets generated or analyzed during this study.
